# A Resource for the Allele-Specific Analysis of DNA Methylation at Multiple Genomically Imprinted Loci in Mice

**DOI:** 10.1534/g3.117.300417

**Published:** 2017-11-14

**Authors:** Jadiel A. Wasson, Onur Birol, David J. Katz

**Affiliations:** * Department of Molecular and Cellular Biology, Harvard University, Cambridge, Massachusetts 02138; † Department of Cell Biology, Emory University School of Medicine, Atlanta, Georgia 30322

**Keywords:** bisulfite analysis, DNA methylation, imprinting, mouse, single nucleotide polymorphism

## Abstract

Genomically imprinted loci are expressed mono-allelically, dependent upon the parent of origin. Their regulation not only illuminates how chromatin regulates gene expression but also how chromatin can be reprogrammed every generation. Because of their distinct parent-of-origin regulation, analysis of imprinted loci can be difficult. Single nucleotide polymorphisms (SNPs) are required to accurately assess these elements allele specifically. However, publicly available SNP databases lack robust verification, making analysis of imprinting difficult. In addition, the allele-specific imprinting assays that have been developed employ different mouse strains, making it difficult to systemically analyze these loci. Here, we have generated a resource that will allow the allele-specific analysis of many significant imprinted loci in a single hybrid strain of *Mus musculus*. This resource includes verification of SNPs present within 10 of the most widely used imprinting control regions and allele-specific DNA methylation assays for each gene in a C57BL/6J and CAST/EiJ hybrid strain background.

Genomically imprinted loci, which are expressed mono-allelically dependent upon their parent of origin, highlight how DNA methylation and chromatin structure can regulate gene expression (Bartolomei and Ferguson-Smith 2011). For example, many of the chromatin mechanisms that regulate imprinted loci are involved in other contexts, including cancer biology and stem cell reprogramming. In addition, alterations at multiple imprinted loci can be used as a readout of global epigenetic misregulation. As a result, there is an increasing need to assay multiple imprinted loci in different mouse models. In this resource article, we provide a streamlined resource for assaying the methylation status of a number of the most studied imprinted genes in a single hybrid strain background.

To date, ∼150 imprinted genes have been identified in mice and ∼100 in humans (Gregg *et al.* 2010; DeVeale *et al.* 2012; Kelsey and Bartolomei 2012). These genes tend to be organized on chromosomes in clusters (Wan and Bartolomei 2008; Bartolomei 2009). This clustering allows multiple imprinted loci to be regulated together, under the control of *cis*-regulatory domains termed imprinting control regions (ICRs) (Wan and Bartolomei 2008; Bartolomei 2009). ICRs are typically between 100 and 3700 bp long and are rich in CpG dinucleotides (Bartolomei and Tilghman 1997; Barlow 2011; Ferguson-Smith 2011). In mammals, DNA methylation occurs mainly in the context of CpG dinucleotides, and within ICRs these CpG dinucleotides are differentially methylated, dependent upon the parent of origin (Reik and Dean 2001; Reik and Walter 2001). This differential methylation determines the expression status of the multiple imprinted genes located within the imprinting cluster (Reik and Walter 2001). Therefore, to globally interrogate the epigenetic control of genomically imprinted loci in a particular mouse model, it is necessary to be able to assay the DNA methylation status of multiple ICRs allele specifically.

Assessing ICRs allele specifically requires taking advantage of single nucleotide polymorphisms (SNPs). C57BL/6J (hereafter referred to as B6) mice are the most commonly used strain of *Mus musculus domesticus* and were the first mouse strain to be fully sequenced (Beck *et al.* 2000). To generate hybrids with SNPs on each allele, B6 mice can be crossed to *M. musculus castaneus* (hereafter referred to as CAST) mice, which originate from a well-defined subgroup of wild mice (Beck *et al.* 2000). Genome-wide DNA sequence analysis between different strains of *M. musculus* revealed a 50% allelic difference between B6 and CAST at potential SNPs (Frazer *et al.* 2007). This makes these hybrid progeny especially useful for analyzing imprinted loci.

SNPs between B6 and CAST are cataloged in the database of SNPs (dbSNP) (https://www.ncbi.nlm.nih.gov/projects/SNP/) (Smigielski *et al.* 2000; Sherry *et al.* 2001). This database reports SNPs that have been observed in various assays performed by individual researchers, consortiums, and genome sequencing centers, for the purpose of facilitating genome-wide association studies (Smigielski *et al.* 2000; Sherry *et al.* 2001). Unfortunately, this database is phasing out all nonhuman organism data by September 2017. However, very similar information will still be housed in the European variation archive (http://www.ebi.ac.uk/eva/?Home). This database overlaps with the dbSNP database and also the Sanger SNP viewer database (https://www.sanger.ac.uk/sanger/Mouse_SnpViewer/rel-1505) (Keane *et al.* 2011; Yalcin *et al.* 2011), which provides SNP information in multiple different strain backgrounds.

Using SNPs from all of these databases, we sought to develop allele-specific DNA methylation assays at multiple ICRs in a B6/CAST hybrid background. However, we encountered two significant hurdles. First, since the dbSNP database and the European variation archive are public repositories, many reported SNPs have not been additionally verified (Mitchell *et al.* 2004; Nekrutenko and Taylor 2012). Moreover, they currently have no minimum requirements for allelic frequencies (Mitchell *et al.* 2004; Nekrutenko and Taylor 2012). This further contributes to the lack of verification for many SNPs. As a result, false positives have been reported at a rate of between 15 and 17% (Mitchell *et al.* 2004; Nekrutenko and Taylor 2012). In addition, these two databases pool sequence differences from different strains into one combined output. Thus, we discovered that relying solely on the dbSNP database or European variation archive leads to an even higher rate of false positives within ICRs. These hurdles can partially be overcome by also incorporating the Sanger database, which contains information from individual strain backgrounds. However, a drawback of the Sanger database is that it contains much less information on intergenic regions, where many ICRs are found. For example, it contains no information on three of the ICRs that we sought to interrogate. In the end, we assessed 93 B6/CAST SNPs from the three databases at 10 of the most commonly studied mouse ICRs, and were able to validate only 18 of them (19%).

The second hurdle that we encountered is the generation of bisulfite PCR assays within ICRs. The gold standard in probing the DNA methylation status of any locus is bisulfite analysis (Hayatsu *et al.* 2008; Laird 2010). As bisulfite analysis relies on detecting base pair changes at CpG dinucleotides, primer sets used for bisulfite PCR cannot contain any CpG dinucleotides because of the uncertainty of whether a cytosine base in the primer-annealing sequence may be methylated. As a result, generating bisulfite-specific primer sets in these highly CpG-rich ICR regions can be difficult. In addition, because the CpG-rich ICRs tend to be repetitive, finding primer sets that amplify a unique product can also be challenging.

Based on the significant hurdles we encountered, we identified a need for optimized protocols for allele-specific DNA methylation analysis of ICRs in a B6/CAST hybrid mouse background. As a result, we developed a resource that includes verification of SNPs present in ICRs, primer information, and optimal PCR conditions. This resource will enable the systematic interrogation of many significant imprinted genes in different mouse models.

## Materials and Methods

### Bisulfite analysis and bisulfite PCR optimization

Mouse tail DNA from single C57BL/6J and CAST/EiJ animals was used for the original identification of SNPs. Subsequently, DNA from sagittal sections of perinatal pups was used for allele-specific DNA methylation analysis. Bisulfite conversion was done according to the Zymo EZ DNA Methylation Kit (Zymo D5001) protocol from 400 ng of DNA. PCR products were amplified in a 15-µl reaction and 3 µl was saved for subsequent TA cloning using the standard TOPO TA cloning protocol (K4500J10; ThermoFisher). The remaining volume was run on a 1% agarose gel to confirm that there is a single PCR product. Bisulfite primers were optimized on bisulfite-converted DNA using 12 different conditions, including four different concentrations of MgCl_2_ (1.5, 2.5, 3.5, and 4.5 mM) paired with three different concentrations of DMSO (0, 1.5, and 5%). In addition, primers were optimized across a temperature gradient. Primer sets, polymorphisms, and optimal PCR conditions for each gene are listed in the individual figures. Of note, because of the difficulty in finding primer sequences in highly CpG-rich regions that do not contain a CpG dinucleotide, many of the primers contained suboptimal base composition and/or did not match the annealing temperature of the other primer used in the reaction. As a result, several of the optimized PCR protocols contain relatively large numbers of cycles to enable the amplification of a product. The BiQ Analyzer program was used for the analysis of bisulfite-converted sequences. During the bisulfite analysis, depending on the choice of primers, two different DNA strands will lead to two different sequencing results. Some of the genes we report here were surveyed on the opposite strand of the gene assembly and therefore have a reversed order of their SNPs compared to the databases. These genes are shown with their chromosome location number in reverse order, from high to low, and this is noted in the corresponding figure legend.

### Data availability

The authors affirm that all data necessary for confirming the results in the article are present in the article. Reagents are available upon request.

## Results

**Figure 1:**
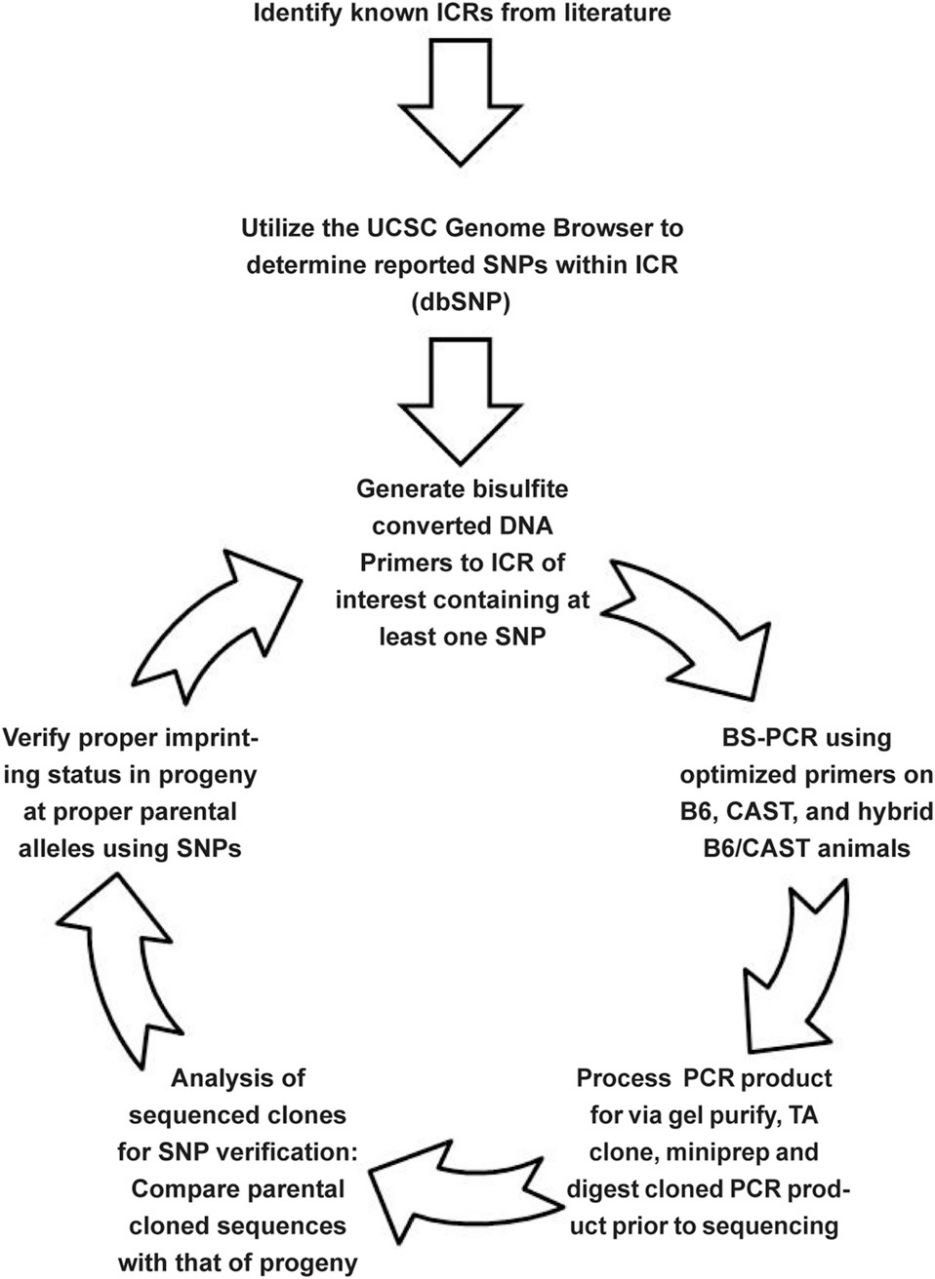
Workflow for SNP verification within ICRs. Known ICRs were first pulled from literature followed by identification of putative SNPs present within each region. These SNPs then underwent a verification process through bisulfite analysis of both parental and hybrid progeny strains. SNPs that fail to verify were fed back to the verification process.

To begin the process of interrogating specific imprinted loci, we generated a workflow to streamline the process (Figure 1). Our first criterion was to identify well-defined ICRs that have been extensively studied. We focused on the following ICRs due to their prevalence in the literature: *Grb10*, *H19*, *Igf2r*, *Impact*, *Lit1/Kcnq1ot1*, *Mest/Peg1*, *Peg3*, *Peg10*, *Snrpn*, and *Zac1/Plagl1*. These ICRs also had well-defined locations in the genome and are associated with differentially methylated regions that allowed us to probe their methylation status via bisulfite analysis.

We then used the UCSC Genome Browser in conjunction with dbSNP to determine reported SNPs within a 10-kb window surrounding and including the ICRs, and these SNPs were then cross-checked against the European database as well as the Sanger database to determine their presence in specific strain backgrounds. Following this *in silico* analysis, we designed bisulfite-specific primers to the regions of interest (Table 1). These regions were <1 kb and were within our 10-kb defined window, including a significant portion of the ICR and at least one SNP. The bisulfite primers could not contain any CpG dinucleotides, reducing the availability of genomic regions to amplify. Bisulfite primers were optimized on bisulfite-converted DNA (detailed in *Materials and Methods*). After optimization, bisulfite PCR was performed on a B6 female and a CAST male, along with the hybrid progeny resulting from the mating. Reported SNPs were compared in B6 and CAST sequences. If validated in this initial comparison, further validation was performed via analysis of the methylation status in hybrid B6/CAST progeny.

**Table 1:** Primer sequences

Gene	DNA Sequence 5′→3′	Bisulfite Converted Sequence 5′→3′
*Grb10*	F-GAGAAAAAAGGTTCAGTTACCCCAG(A/G)	F-GAGAAAAAAGGTTTAGTTATTTTAG(A/G)
	R-CCTCCCGAAATCTGCAATGGTC	R-CCTCCCAAAATCTACAATAATC
*H19*	F-ATTCACAAATGGCAATGCTGTGG	F-ATTTATAAATGGTAATGTTGTGG
	R-CCTCATGAAGCCCATGACTAT	R-CCTCATAAAACCCATAACTAT
*Igf2r*	F-CAGAGGATTTTAGCACAACTCCAA	F-TAGAGGATTTTAGTATAATTTTAA
	R-CACTTTTGAGCTTGCCTCTCTTGC	R-CACTTTTGAGCTTGCCTCTCTTGC
*Impact*	F-CTGCATAGTTTTGCTCTCATAAGTG	F-TTGTATAGTTTTGTTTTTATAAGTG
	R-GGCCTGCTCATGTGACAATGCGGC	R-AACCTACTCATATAACAATACAAC
*Lit1*	F-CAAGGTGAGTGGCCTAGGAC	F-TAAGGTGAGTGGTTTAGGAT
	R-AATCCCCCACACCTGAATTC	R-AATCCCCCACACCTAAATTC
*Mest*	F-GGGTGTTTTATGTCTTCCAGGG(T/G)	F-GGGTGTTTTATGTTTTTTAGGG(T/G)
	R-CCCAGATTCTAGTGAAGAAAGCCTTCCCAT	R-CCCAAATTCTAATAAAAAAAACCTTCCCAT
*Peg3*	F-GGTGCATCTTTACTGCCAACTAGCAAAG	F-GGTGTATTTTTATTGTTAATTAGTAAAG
	R-CAGGTTTGCTGCACAGGCTTATCC	R-CAAATTTACTACACAAACTTATCC
*Peg10*	F-GCAAAGTGACTGGCTCTGCACTCTTAAGTG	F-GTAAAGTGATTGGTTTTGTATTTTTAAGTG
	R-TTGGTTACTCTCCTGCAGCTTTCCAAATT	R-TTAATTACTCTCCTACAACTTTCCAAATT
*Snrpn*	F-GCAATTATATCCATTATTCCAGATTGACAGTGA(T/G)	F-GTAATTATATTTATTATTTTAGATTGATAGTGA(T/G)
	R-ATAGGATGCACTTTCACTACTAGAATCC	R-ATAAAATACACTTTCACTACTAAAATCC
*Zac1*	F-GGGTAGGTAAGTAGTGACAA	F-GGGTAGGTAAGTAGTGATAA
	R-CCTAAAACACCAAAGTAGCA	R-CCTAAAACACCAAAATAACA

Using this workflow, we validated SNPs in all 10 ICRs and identified PCR conditions for the analysis of each. The relevant details are reported for each gene below. In addition, we have shown each single band amplicon run in an agarose gel (Supplemental Material, Figure S1).

### Grb10

**Figure 2:**
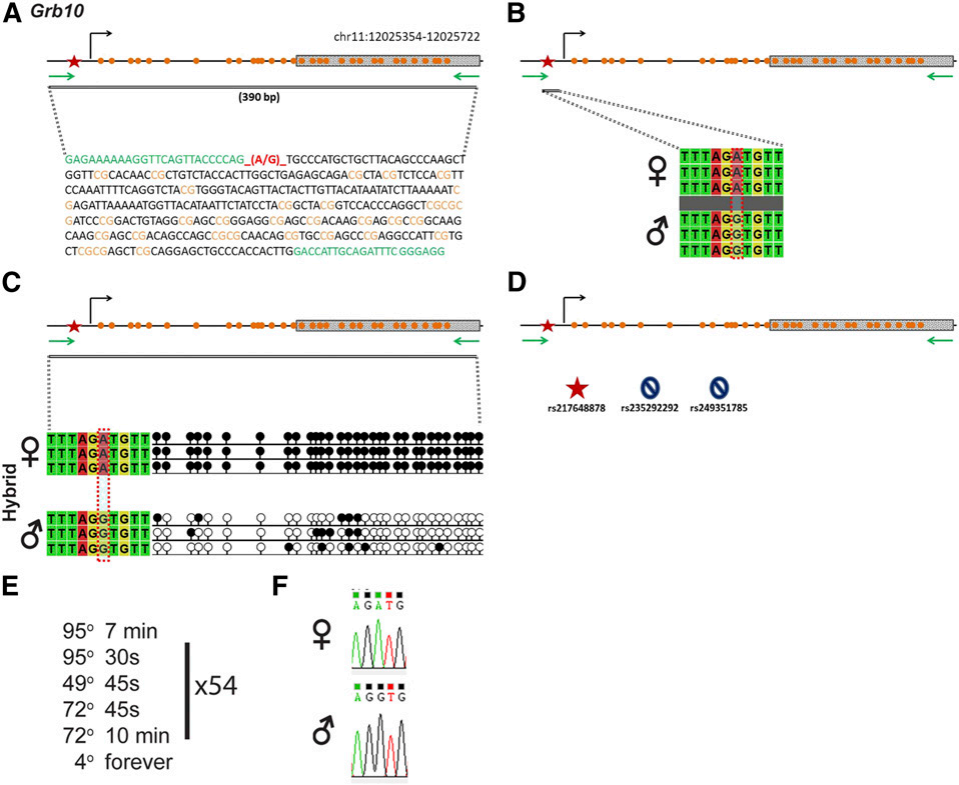
SNP verification within Grb10 ICR. (A) Schematic of Grb10 ICR. Probed region is highlighted by double-dashed line with number of base pairs covered reported. CpG island indicated by dotted box. Green indicates primer sequences; orange indicates CpG dinucleotides; red star and bases indicate verified SNP. (B) Verified SNP presented as sequences from B6 female and CAST male. A-to-G SNP is highlighted by red dotted rectangle. (C) Verification of proper imprinted status in hybrid B6/CAST progeny. SNP highlighted by red dotted rectangle. DNA methylation presented as lollipop diagram; White circles indicate unmethylated cytosines; black circles indicate methylated cytosines. (D) Other SNPs reported in all three databases within the probed region with the SNP highlighted by red dotted rectangle. dbSNP identification number indicated under each SNP. Red star indicates validated SNP and blue closed circle indicates C-to-T polymorphism that cannot be assayed in bisulfite analysis. (E) Optimal PCR conditions for probed region with the given primers. (F) The electropherogram indicating A-to-G polymorphism for the SNP region. ♀, maternal allele; ♂, paternal allele.

*Grb10* is regulated by an ICR that is ∼1.4 kb and located on chromosome 11 in mouse (Figure 2A). Within our probed region, we validated one SNP out of three reported SNPs from the dbSNP database (Figure 2D). The validated SNP is within a 390-bp region containing 31 CpG residues (Figure 2A), with the polymorphic base being an A in the B6 background and a G in the CAST background (Figure 2B). *Grb10* is methylated on the maternal allele and unmethylated on the paternal allele. This methylation pattern was correctly observed in the hybrid progeny using our optimized assay (Figure 2, C and E).

### H19

**Figure 3:**
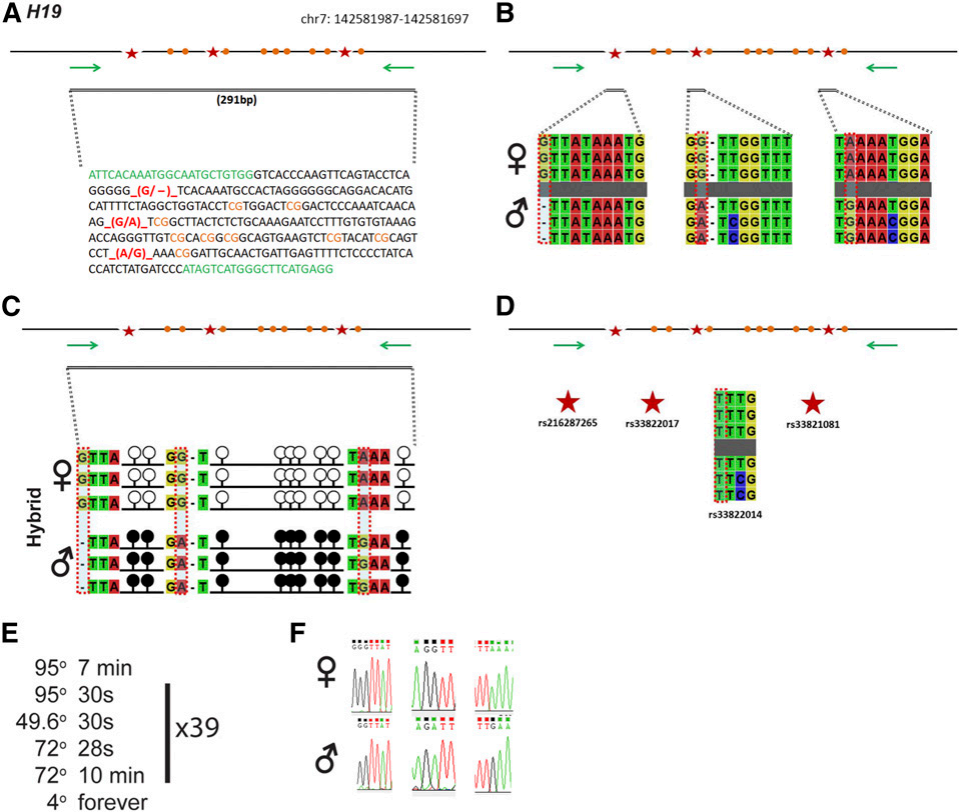
SNP verification within H19 ICR. (A) Schematic of H19 ICR. Probed region is highlighted by double-dashed line with number of base pairs covered reported. CpG island indicated by dotted box. Green indicates primer sequences; orange indicates CpG dinucleotides; red star and bases indicate verified SNPs. The chromosome location is from high to low, see *Materials and Methods* for more details. (B) Verified SNPs presented as sequences from B6 female and CAST male. G-to-del, G-to-A, and A-to-G SNPs are highlighted by red dotted rectangle. (C) Verification of proper imprinted status in hybrid B6/CAST progeny. SNPs highlighted by red dotted rectangle. DNA methylation presented as lollipop diagram; white circles indicate unmethylated cytosines; black circles indicate methylated cytosines. (D) Other SNPs reported in all three databases within the probed region with the SNP highlighted by red dotted rectangle. dbSNP identification number indicated under each SNP. Red star indicates validated SNP and blue closed circle indicates C-to-T polymorphism that cannot be assayed in bisulfite analysis. (E) Optimal PCR conditions for probed region with the given primers. (F) The electropherograms indicating G-to-del, G-to-A, and A-to-G polymorphisms for the SNPs. ♀, maternal allele; ♂, paternal allele.

*H19* is regulated by an ICR on chromosome 7 (Figure 3A). Within our probed region, we validated three SNPs out of four reported SNPs from the dbSNP database (Figure 3D). These validated SNPs are within a 291-bp region containing nine CpG residues (Figure 3A). The three validated SNPs include (1) a G in the B6 background and a deletion in the CAST background, (2) a G in the B6 background and an A in the CAST background, and (3) an A in the B6 background and a G in the CAST background (Figure 3B). *H19* is methylated on the paternal allele and unmethylated on the maternal allele. This methylation pattern was correctly observed in the hybrid progeny using our optimized assay (Figure 3, C and E).

### Igf2r

**Figure 4:**
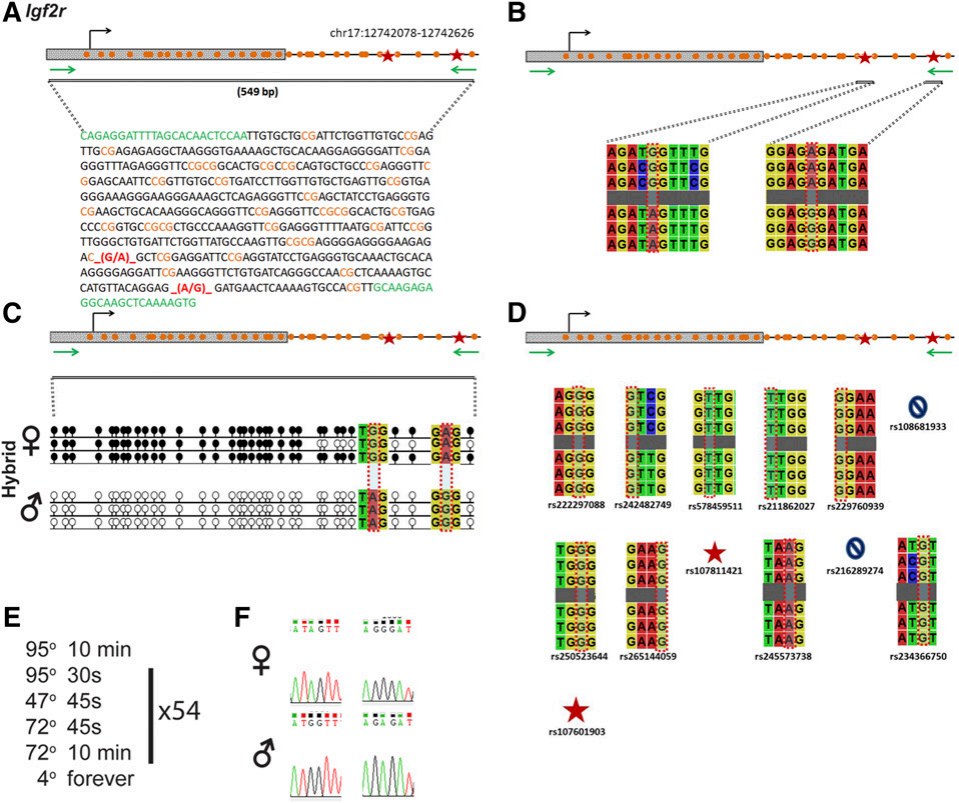
SNP verification within Igf2r ICR. (A) Schematic of Igf2r ICR. Probed region is highlighted by double-dashed line with number of base pairs covered reported. CpG island indicated by dotted box. Green indicates primer sequences; orange indicates CpG dinucleotides; red star and bases indicate verified SNPs. (B) Verified SNPs presented as sequences from B6 female and CAST male. G-to-A and A-to-G SNPs are highlighted by red dotted rectangle. (C) Verification of proper imprinted status in hybrid B6/CAST progeny. SNPs highlighted by red dotted rectangle. DNA methylation presented as lollipop diagram; white circles indicate unmethylated cytosines; black circles indicate methylated cytosines. (D) Other SNPs reported in all three databases within the probed region with the SNP highlighted by red dotted rectangle. dbSNP identification number indicated under each SNP. Red star indicates validated SNP and blue closed circle indicates C-to-T polymorphism that cannot be assayed in bisulfite analysis. (E) Optimal PCR conditions for probed region with the given primers. (F) The electropherograms indicating G-to-A and A-to-G polymorphisms for the SNP regions. ♀, maternal allele; ♂, paternal allele.

*Igf2r* is regulated by an ICR on chromosome 17 (Figure 4A). Within our probed region, we validated two SNPs out of 13 reported SNPs from the dbSNP database (Figure 4D). These validated SNPs are within a 549-bp region containing 33 CpG residues (Figure 4A). These polymorphic bases include (1) a G in the B6 background and an A in the CAST background, and (2) an A in the B6 background and a G in the CAST background (Figure 4B). *Igf2r* is methylated on the maternal allele and unmethylated on the paternal allele. This methylation pattern was correctly observed in the hybrid progeny using our optimized assay (Figure 4 C and E).

### Impact

**Figure 5:**
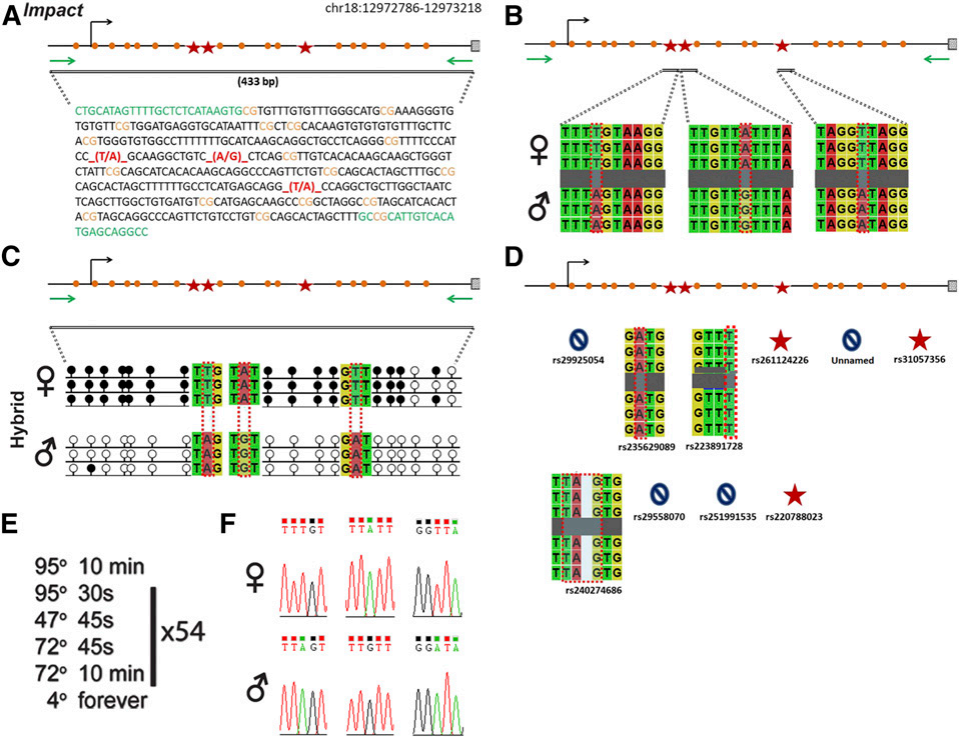
SNP verification within Impact ICR. (A) Schematic of Impact ICR. Probed region is highlighted by double-dashed line with number of base pairs covered reported. CpG island indicated by dotted box. Green indicates primer sequences; orange indicates CpG dinucleotides; red star and bases indicate verified SNPs. (B) Verified SNPs presented as sequences from B6 female and CAST male. T-to-A, A-to-G, and T-to-A SNPs are highlighted by red dotted rectangle. (C) Verification of proper imprinted status in hybrid B6/CAST progeny. SNPs highlighted by red dotted rectangle. DNA methylation presented as lollipop diagram; white circles indicate unmethylated cytosines; black circles indicate methylated cytosines. (D) Other SNPs reported in all three databases within the probed region with the SNP highlighted by red dotted rectangle. dbSNP identification number indicated under each SNP. Red star indicates validated SNP and blue closed circle indicates C-to-T polymorphism that cannot be assayed in bisulfite analysis. (E) Optimal PCR conditions for probed region with the given primers. (F) The electropherograms indicating T-to-A, A-to-G, and T-to-A polymorphisms for the SNP regions. ♀, maternal allele; ♂, paternal allele.

*Impact* is regulated by an ICR on chromosome 18 (Figure 5A). Within our probed region, we validated three SNPs out of 10 reported SNPs from the dbSNP and European databases (Figure 5D). One of the SNPs that was not validated was an unnamed SNP from the European database. The validated SNPs are within a 433-bp region that contains 17 CpG residues (Figure 5A). These polymorphic bases include (1) a T in the B6 background and an A in the CAST background, (2) an A in the B6 background and a G in the CAST background, and (3) a T in the B6 background and an A in the CAST background (Figure 5B). *Impact* is methylated on the maternal allele and unmethylated on the paternal allele. This methylation pattern was correctly observed in the hybrid progeny using our optimized assay (Figure 5, C and E).

### Lit1/Kcnq1ot1

**Figure 6:**
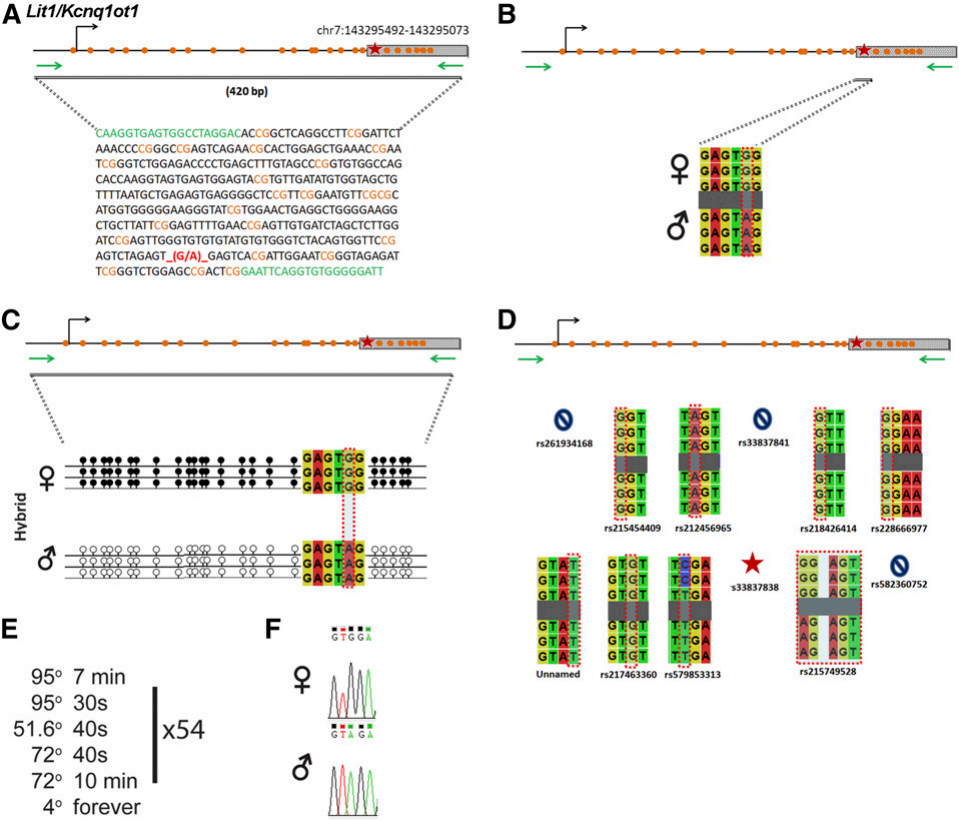
SNP verification within Lit1/Kcnq1ot1 ICR. (A) Schematic of Lit1/Kcnq1ot1 ICR. Probed region is highlighted by double-dashed line with number of base pairs covered reported. CpG island indicated by dotted box. Green indicates primer sequences; orange indicates CpG dinucleotides; red star and bases indicate verified SNP. The chromosome location is from high to low, see *Materials and Methods* for more details. (B) Verified SNP presented as sequences from B6 female and CAST male. G-to-A SNP is highlighted by red dotted rectangle. (C) Verification of proper imprinted status in hybrid B6/CAST progeny. SNP highlighted by red dotted rectangle. DNA methylation presented as lollipop diagram; white circles indicate unmethylated cytosines; black circles indicate methylated cytosines. (D) Other SNPs reported in all three databases within the probed region with the SNP highlighted by red dotted rectangle. dbSNP identification number indicated under each SNP. Red star indicates validated SNP and blue closed circle indicates C-to-T polymorphism that cannot be assayed in bisulfite analysis. (E) Optimal PCR conditions for probed region with the given primers. (F) The electropherogram indicating G-to-A polymorphism for the SNP region. ♀, maternal allele; ♂, paternal allele.

*Lit1/Kcnq1ot1* is regulated by an ICR on chromosome 7 (Figure 6A). Within our probed region, we validated one SNP out of 12 reported SNPs from the dbSNP and European databases (Figure 6D). One of the SNPs that was not validated was an unnamed SNP from the European database. The validated SNP is within a 420-bp region that contains 17 CpG residues (Figure 6A). The polymorphic base is a G in the B6 background and an A in the CAST background (Figure 6B). *Lit1* is methylated on the maternal allele and unmethylated on the paternal allele. This methylation pattern was correctly observed in the hybrid progeny using our optimized assay (Figure 6, C and E).

### Mest/Peg1

**Figure 7:**
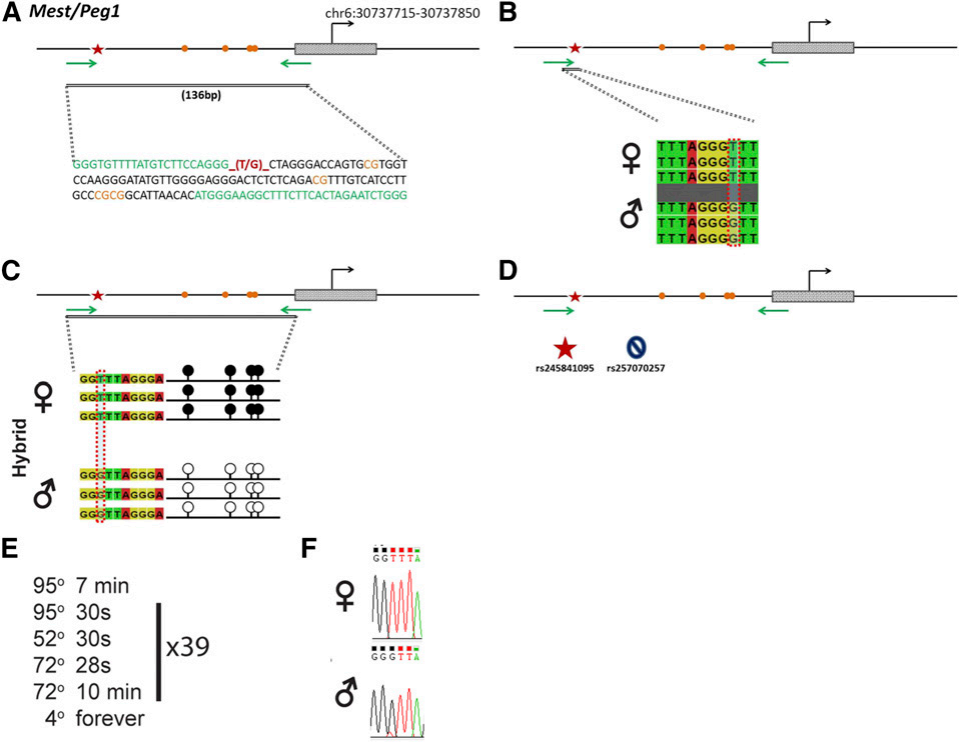
SNP verification within Mest/Peg1 ICR. (A) Schematic of Mest/Peg1 ICR. Probed region is highlighted by double-dashed line with number of base pairs covered reported. CpG island indicated by dotted box. Green indicates primer sequences; orange indicates CpG dinucleotides; red star and bases indicate verified SNP. (B) Verified SNP presented as sequences from B6 female and CAST male. T-to-G SNP is highlighted by red dotted rectangle. (C) Verification of proper imprinted status in hybrid B6/CAST progeny. SNP highlighted by red dotted rectangle. DNA methylation presented as lollipop diagram; white circles indicate unmethylated cytosines; black circles indicate methylated cytosines. (D) Other SNPs reported in all three databases within the probed region with the SNP highlighted by red dotted rectangle. dbSNP identification number indicated under each SNP. Red star indicates validated SNP and blue closed circle indicates C-to-T polymorphism that cannot be assayed in bisulfite analysis. (E) Optimal PCR conditions for probed region with the given primers. (F) The electropherogram indicating T-to-G polymorphism for the SNP region. ♀, maternal allele; ♂, paternal allele.

*Mest/Peg1* is regulated by an ICR on chromosome 6 (Figure 7A). Within our probed region, we validated one SNP out of two reported SNPs from the dbSNP database (Figure 7D). This validated SNP is within a 136-bp region that contains four CpG residues (Figure 7A). This polymorphic base is a T in the B6 background and a G in the CAST background (Figure 7B). *Mest* is methylated on the maternal allele and unmethylated on the paternal allele. This methylation pattern was correctly observed in the hybrid progeny using our optimized assay (Figure 7, C and E).

### Peg3

**Figure 8:**
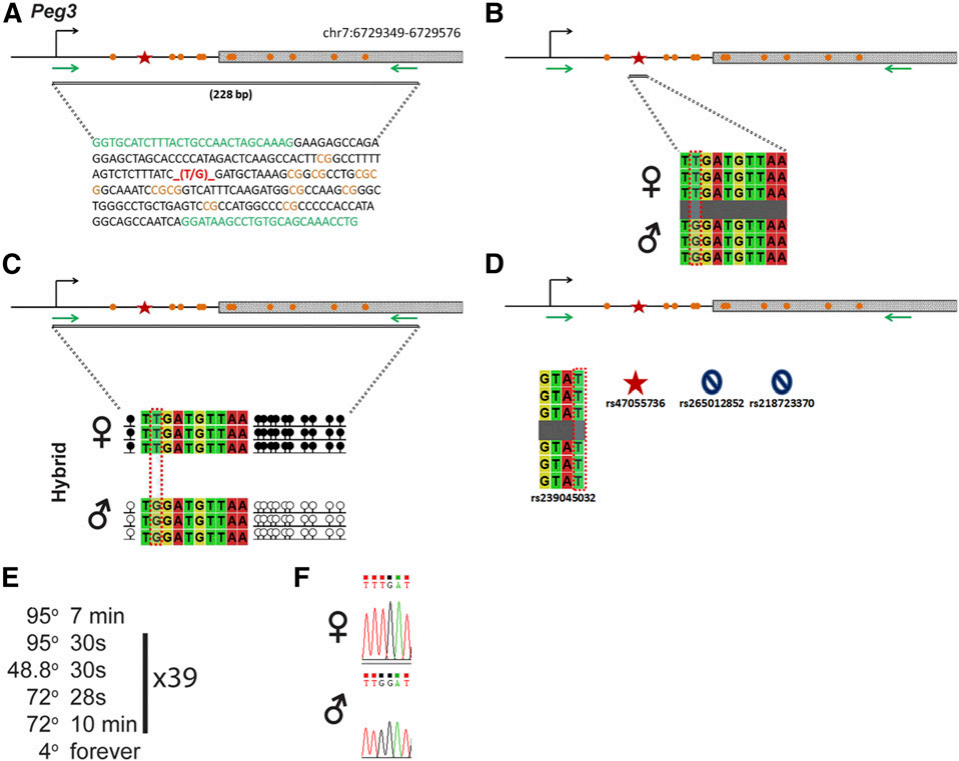
SNP verification within Peg3 ICR. (A) Schematic of Peg3 ICR. Probed region is highlighted by double-dashed line with number of base pairs covered reported. CpG island indicated by dotted box. Green indicates primer sequences; orange indicates CpG dinucleotides; red star and bases indicate verified SNP. (B) Verified SNP presented as sequences from B6 female and CAST male. T-to-G SNP is highlighted by red dotted rectangle. (C) Verification of proper imprinted status in hybrid B6/CAST progeny. SNP highlighted by red dotted rectangle. DNA methylation presented as lollipop diagram; white circles indicate unmethylated cytosines; black circles indicate methylated cytosines. (D) Other SNPs reported in all three databases within the probed region with the SNP highlighted by red dotted rectangle. dbSNP identification number indicated under each SNP. Red star indicates validated SNP and blue closed circle indicates C-to-T polymorphism that cannot be assayed in bisulfite analysis. (E) Optimal PCR conditions for probed region with the given primers. (F) The electropherogram indicating the T-to-G polymorphism for the SNP region. ♀, maternal allele; ♂, paternal allele.

*Peg3* is regulated by an ICR on chromosome 7 (Figure 8A). Within our probed region, we validated one SNP out of four reported SNPs from the dbSNP database (Figure 8D). This validated SNP is within a 228-bp region that contains 11 CpG residues (Figure 8A). This polymorphic base is a T in the B6 background and a G in the CAST background (Figure 8B). *Peg3* is methylated on the maternal allele and unmethylated on the paternal allele. This methylation pattern was correctly observed in the hybrid progeny using our optimized assay (Figure 8, C and E).

### Peg10

**Figure 9:**
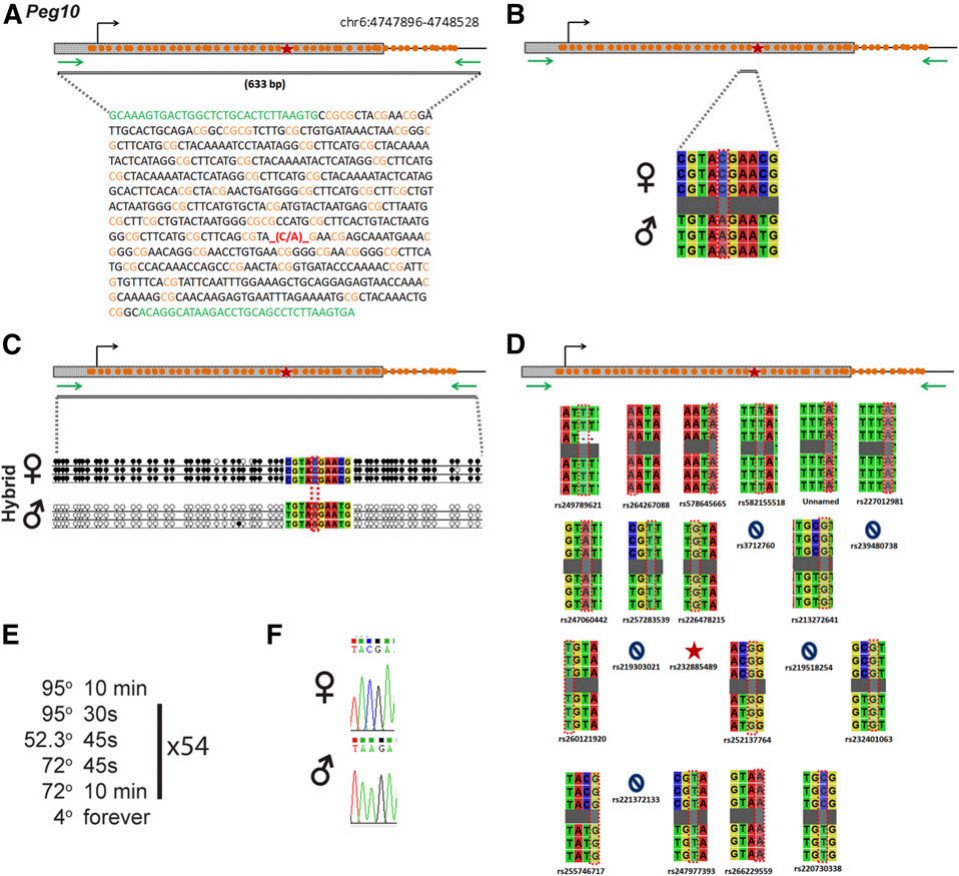
SNP verification within Peg10 ICR. (A) Schematic of Peg10 ICR. Probed region is highlighted by double-dashed line with number of base pairs covered reported. CpG island indicated by dotted box. Green indicates primer sequences; orange indicates CpG dinucleotides; red star and bases indicate verified SNP. (B) Verified SNP presented as sequences from B6 female and CAST male. C-to-A SNP is highlighted by red dotted rectangle. (C) Verification of proper imprinted status in hybrid B6/CAST progeny. SNP highlighted by red dotted rectangle. DNA methylation presented as lollipop diagram; white circles indicate unmethylated cytosines; black circles indicate methylated cytosines. (D) Other SNPs reported in all three databases within the probed region with the SNP highlighted by red dotted rectangle. dbSNP identification number indicated under each SNP. Red star indicates validated SNP and blue closed circle indicates C-to-T polymorphism that cannot be assayed in bisulfite analysis. (E) Optimal PCR conditions for probed region with the given primers. (F) The electropherogram indicating C-to-A polymorphism for the SNP region. ♀, maternal allele; ♂, paternal allele.

*Peg10* is regulated by an ICR on chromosome 6 (Figure 9A). Within our probed region, we validated one SNP out of 23 reported SNPs from the dbSNP and European databases (Figure 9D). One of the SNPs that was not validated was an unnamed SNP from the European database. The validated SNP is within a 663-bp region that contains 54 CpG residues (Figure 9A). This polymorphic base is a C in the B6 background and an A in the CAST background (Figure 9B). *Peg10* is methylated on the maternal allele and unmethylated on the paternal allele. This methylation pattern was correctly observed in the hybrid progeny using our optimized assay (Figure 9, C and E).

### Snrpn

**Figure 10:**
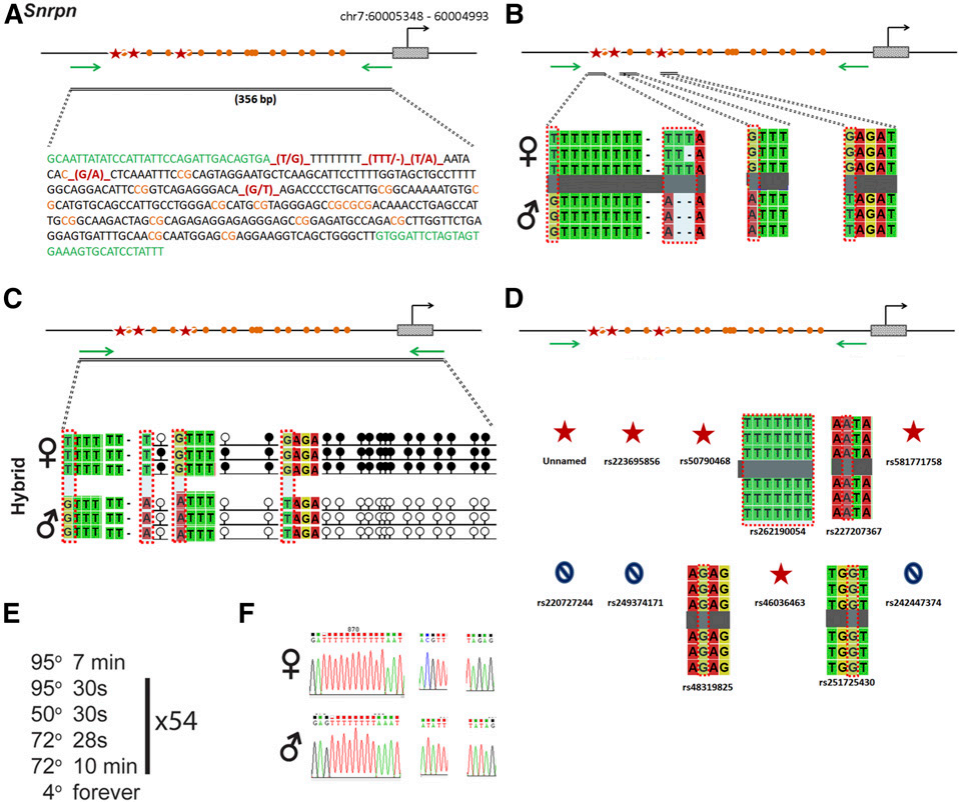
SNP verification within Snrpn ICR. (A) Schematic of Snrpn ICR. Probed region is highlighted by double-dashed line with number of base pairs covered reported. CpG island indicated by dotted box. Green indicates primer sequences; orange indicates CpG dinucleotides; red star and bases indicate verified SNPs. The chromosome location is from high to low, see *Materials and Methods* for more details. (B) Verified SNPs presented as sequences from B6 female and CAST male. T-to-G, TTT-to-del, T-to-A, G-to-A, and G-to-T SNPs are highlighted by red dotted rectangle. (C) Verification of proper imprinted status in hybrid B6/CAST progeny. SNP highlighted by red dotted rectangle. DNA methylation presented as lollipop diagram; white circles indicate unmethylated cytosines; black circles indicate methylated cytosines. (D) Other SNPs reported in all three databases within the probed region with the SNP highlighted by red dotted rectangle. dbSNP identification number indicated under each SNP. Red star indicates validated SNP and blue closed circle indicates C-to-T polymorphism that cannot be assayed in bisulfite analysis. (E) Optimal PCR conditions for probed region with the given primers. (F) The electropherograms indicating T-to-G, TTT-to-del, T-to-A, G-to-A, and G-to-T polymorphisms for the SNP regions. ♀, maternal allele; ♂, paternal allele.

*Snrpn* is regulated by an ICR on chromosome 7 (Figure 10A). Within our probed region, we validated four SNPs out of 11 reported SNPs from the dbSNP database (Figure 10D). We also identified a novel SNP that is not present in any of the three databases. All five of the validated SNPs are within a 356-bp region that contains 16 CpG residues (Figure 10A). These polymorphic bases include (1) a T in the B6 background and a G in the CAST background, this is the novel SNP that we identified; (2) a TTT in the B6 background and a deletion in the CAST background; (3) a T in the B6 background and an A in the CAST background; (4) a G in the B6 background and an A in the CAST background; and (5) a G in the B6 background and a T in the CAST background (Figure 10B). *Snrpn* is methylated on the maternal allele and unmethylated on the paternal allele. This methylation pattern was correctly observed in the hybrid progeny using our optimized assay (Figure 10, C and E).

### Zac1/Plagl1

**Figure 11:**
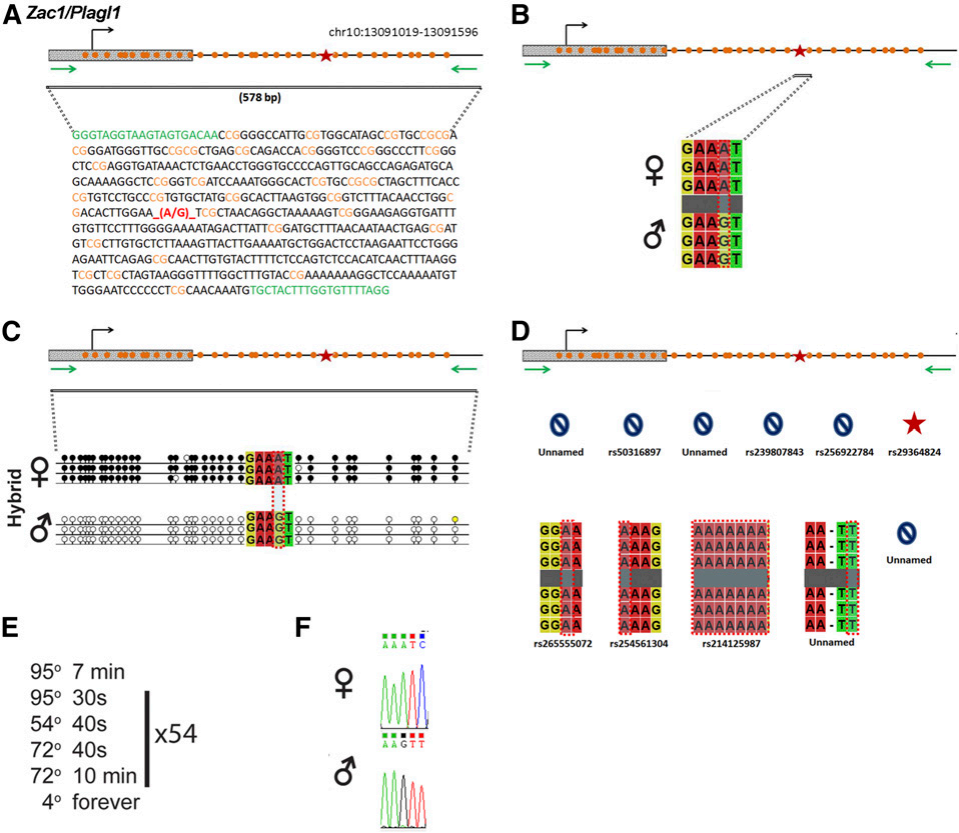
SNP verification within Zac1/Plagl1 ICR. (A) Schematic of Zac1/Plagl1 ICR. Probed region is highlighted by double-dashed line with number of base pairs covered reported. CpG island indicated by dotted box. Green indicates primer sequences; orange indicates CpG dinucleotides; red star and bases indicate verified SNP. (B) Verified SNP presented as sequences from B6 female and CAST male. A-to-G SNP is highlighted by red dotted rectangle. (C) Verification of proper imprinted status in hybrid B6/CAST progeny. SNP highlighted by red dotted rectangle. DNA methylation presented as lollipop diagram; white circles indicate unmethylated cytosines; black circles indicate methylated cytosines. (D) Other SNPs reported in all three databases within the probed region with the SNP highlighted by red dotted rectangle. dbSNP identification number indicated under each SNP. Red star indicates validated SNP and blue closed circle indicates C-to-T polymorphism that cannot be assayed in bisulfite analysis. (E) Optimal PCR conditions for probed region with the given primers. (F) The electropherogram indicating A-to-G polymorphism for the SNP region. ♀, maternal allele; ♂, paternal allele.

*Zac1/Plagl1* is regulated by an ICR on chromosome 10 (Figure 11A). Within our probed region, we validated one SNP out of 11 reported SNPs from the dbSNP and European databases (Figure 11D). The unnamed SNPs are not found in the dbSNP. The validated SNP is within a 578-bp region that contains 33 CpG residues (Figure 11A). This polymorphic base is an A in the B6 background and a G in the CAST background (Figure 11B). *Zac1* is methylated on the maternal allele and unmethylated on the paternal allele. This methylation pattern was correctly observed in the hybrid progeny using our optimized assay (Figure 11, C and E).

## Discussion

Of the SNPs that we analyzed, we were able to validate 18, while we failed to validate 75 SNPs within those same regions (Table 2, red and black). In addition, of those 75 SNPs, 28 of them were C/T polymorphisms that bisulfite analysis was unable to differentiate (Table 2, blue). We also identified a SNP in the Snrpn ICR, which was not present in any of the three databases (Table 2, orange). Furthermore, during our optimization we failed to validate multiple SNPs that lie outside of our bisulfite primers. These SNPs are reported in Figure S2. Among the many SNPs reported in the dbSNP database that we failed to verify, most were identified as SNPs between strains other than CAST in the Sanger database. In the end, we could only find one SNP that was supposed to show a polymorphism based on the reported data but did not in our experiments (Table 2, purple). Thus, in general, we recommend using the Sanger database. However, it is important to note that since the Sanger database primarily contains SNPs located close to or within genes, certain ICR SNPs had to be identified in the dbSNP database.

**Table 2:** The complete list of all the SNPs from 3 databases within surveyed regions

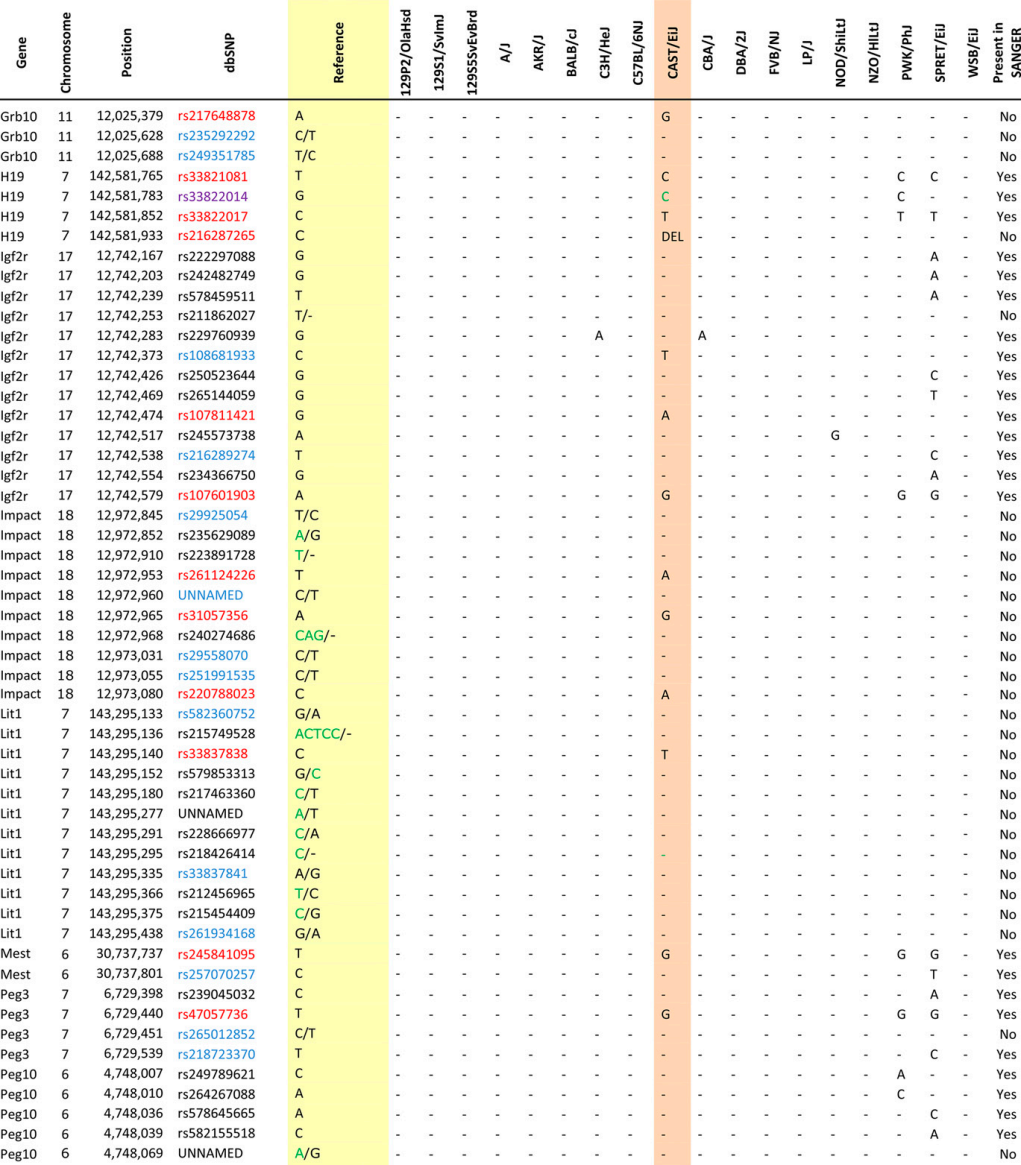 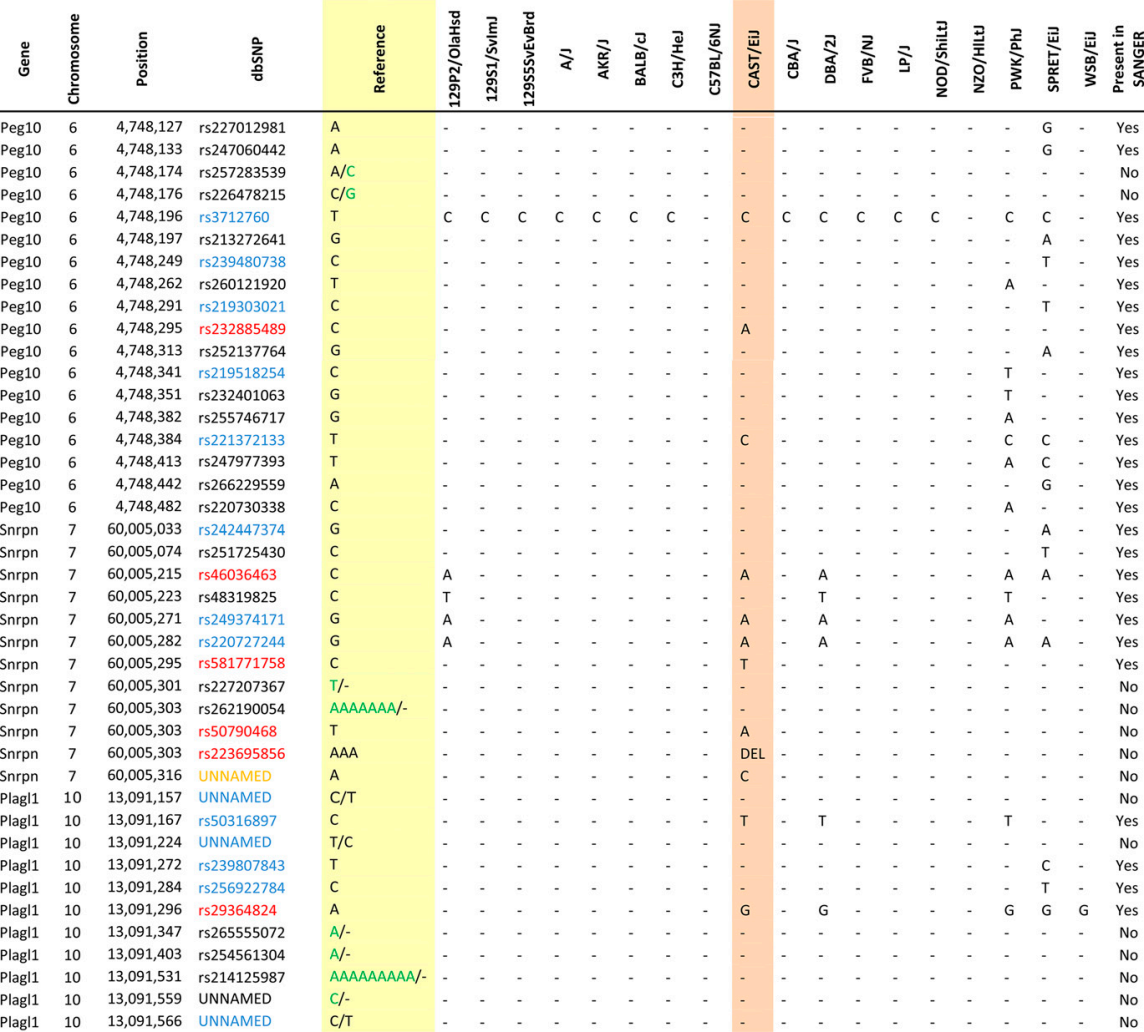

Orange indicates that the SNP is the SNP we have found; it is not present in any database. Red indicates that SNPs are validated polymorphisms. Blue indicates that SNPs are C/T (or G/A) variations that bisulfite sequencing assay can’t detect. Purple indicates that the SNP is the only inconsistency between our sequencing result (C on B6 background) and the reported Sanger data (G on B6 background). Green nucleotides indicate the present polymorphism on both assayed backgrounds (B6 and CAST) at reported SNP locations

In this resource, we have validated a number of SNPs within the ICRs of the most commonly imprinted loci. In addition, we have demonstrated a high frequency of invalid SNPs within ICRs when the pooled SNPs from the dbSNP (European variation archive) are used alone, highlighting the drawbacks of the mixed strain databases compared to the Sanger strain-specific polymorphism database. Using the validated SNPs, we have optimized allele-specific DNA methylation assays that will allow for the rapid analysis of multiple imprinted loci in a variety of contexts, including at several ICRs that are not contained within the Sanger database. This resource will enable the systematic analysis of multiple imprinted genes in a number of potential applications.

### Potential Applications

As this resource offers extensive and straightforward assays to interrogate the most commonly studied imprinted loci, it can be used across a number of fields. There are two major instances where we envision the utility of this resource: (1) cases where a regulatory mechanism directly interacts with multiple imprinted loci and (2) cases where a mechanism either indirectly regulates many imprinted loci or affects multiple imprinted loci by generally disrupting the epigenetic landscape.

Recently, a number of proteins have been demonstrated to directly regulate multiple imprinted loci. These include, but are not limited to, *Dnmt3l*, *Dnmt1*, *Lsd2*, *Trim28*, *Zfp57*, and *Tet1/2*, each with a different mechanism of action (Bourc’his *et al.* 2001; Howell *et al.* 2001; Reik *et al.* 2003; Li *et al.* 2008; Karytinos *et al.* 2009; Fang *et al.* 2010; Messerschmidt *et al.* 2012; Yamaguchi *et al.* 2013; Canovas and Ross 2016). For example, deletion of the regulatory subunit of the *de novo* DNA methyltransferase *Dnmt3L* results in the failure to establish maternal DNA methylation at a number of maternally imprinted loci, including *Peg3*, *Lit1/Kcnq1ot1*, and *Snrpn* (Bourc’his *et al.* 2001; Hata *et al.* 2002). Another maternal effect enzyme required for the establishment of DNA methylation at maternally imprinted loci is the histone demethylase *Lsd2*. Mechanistically, *Lsd2* is required to remove H3K4 methylation to get proper DNA methylation at imprinted loci including *Mest*, *Grb10*, and *Zac1* (Ciccone *et al.* 2009; Fang *et al.* 2010; Zhang *et al.* 2012; Stewart *et al.* 2015). Furthermore, *Zfp57*, a KRAB domain zinc-finger protein, is required both maternally and zygotically to maintain the imprinting status of various imprinted loci including *Snrpn* (Li *et al.* 2008; Strogantsev and Ferguson-Smith 2012; Strogantsev *et al.* 2015). This protein is thought to bind directly to DNA with its zinc fingers and subsequently recruit factors that repress transcription (Li *et al.* 2008; Quenneville *et al.* 2011; Strogantsev *et al.* 2015). These studies demonstrate how disruptions in mechanistically distinct regulatory mechanisms can affect multiple imprinted loci.

Alternatively, a number of mechanisms have been demonstrated to indirectly affect imprinted loci via general epigenetic disruptions. For example, mutations in human NLRP genes, which are required maternally for the transition to zygotic gene expression, result in hydatidiform moles and loss of imprinting (Docherty *et al.* 2015). Another maternal effect gene, *Lsd1*, the homolog of *Lsd2*, is also maternally required at fertilization for the maternal to zygotic transition (Ancelin *et al.* 2016; Wasson *et al.* 2016). Loss of maternal *Lsd1* leads to a general disruption of DNA methylation in the resulting progeny at both maternally and paternally imprinted loci (Ancelin *et al.* 2016; Wasson *et al.* 2016). These studies demonstrate how maternal factors, deposited into the zygote from the mother, are required for proper imprinting and development of the embryo.

As ICRs are inherently asymmetric in their epigenetic modifications and opposing mechanisms are required at each parental ICR, even slight disturbances in the epigenetic landscape can lead to significant changes in expression at these loci. For example, disruptions in the maternal expression of *Grb10* results in developmental defects in mice, while disruption of the paternal allele of *Grb10* leads to changes in behavior, including increased social dominance (Garfield *et al.* 2011; Dent and Isles 2014). This highlights differences in the roles of imprinted parental alleles in mice. Another study that highlights the relative contributions of each parental allele describes parental-specific duplications of the 15q11.2-q13.3 region of human chromosome 15 (Isles *et al.* 2016). Paternal duplications were more associated with autism spectrum disorder and developmental delay, while maternal duplications were more associated with psychiatric disorders (Isles *et al.* 2016). These studies demonstrate the complexity of outcomes associated with maternal *vs.* paternal inheritance.

Finally, mechanisms that affect imprinted genes indirectly though general epigenetic disruptions highlight how the methylation status of ICRs can act as a proxy for global epigenetic alterations. For example, studies have demonstrated hypomethylation of a differentially methylated region in the *Igf2-H19* locus in Wilms tumor patients (Scharnhorst *et al.* 2001). In addition, embryos conceived using artificial reproductive technologies have higher incidences of Prader–Willi and Angelman syndromes (Horsthemke and Wagstaff 2008; Buiting 2010; Butler 2011). These syndromes are caused by large-scale chromosomal abnormalities that affect multiple imprinted loci (Horsthemke and Wagstaff 2008; Buiting 2010; Butler 2011). It is also possible that imprinting may be disrupted by environmental factors. For example, Bisphenol A, an environmental toxin, as well as various endocrine disruptors, have been revealed to significantly alter the epigenetic landscape (Kang *et al.* 2011; Susiarjo *et al.* 2013). Also, Vinclozolin exposure in mice leads to infertility due to sperm defects in mice, which correlates with global alterations in the DNA methylation landscape (Anway *et al.* 2005; Kang *et al.* 2011). These studies demonstrate additional mechanisms that may lead to broad imprinting disruptions.

### Conclusion

Due to various mechanisms that can disrupt the epigenetic landscape, we anticipate a growing need to assay imprinted loci in different mouse models. The resource provided here will facilitate the future analysis of multiple imprinted loci in a single hybrid genetic background.

## Supplementary Material

Supplemental material is available online at www.g3journal.org/lookup/suppl/doi:10.1534/g3.117.300417/-/DC1.
