# Supplementary figures and images for: A Resource for the Allele-Specific Analysis of DNA Methylation at Multiple Genomically Imprinted Loci in Mice
Source: G3 (Bethesda). 2017 Nov 14;8(1):91–103. doi: 10.1534/g3.117.300417 (PMC5765370; doi:10.1534/g3.117.300417)

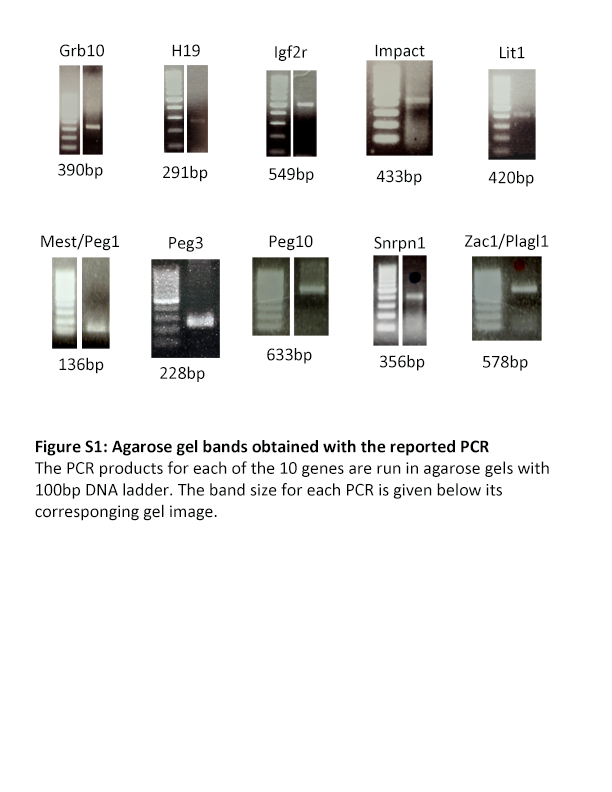

Supplement: Supplementary file 1 [file 91FigureS1.tif]

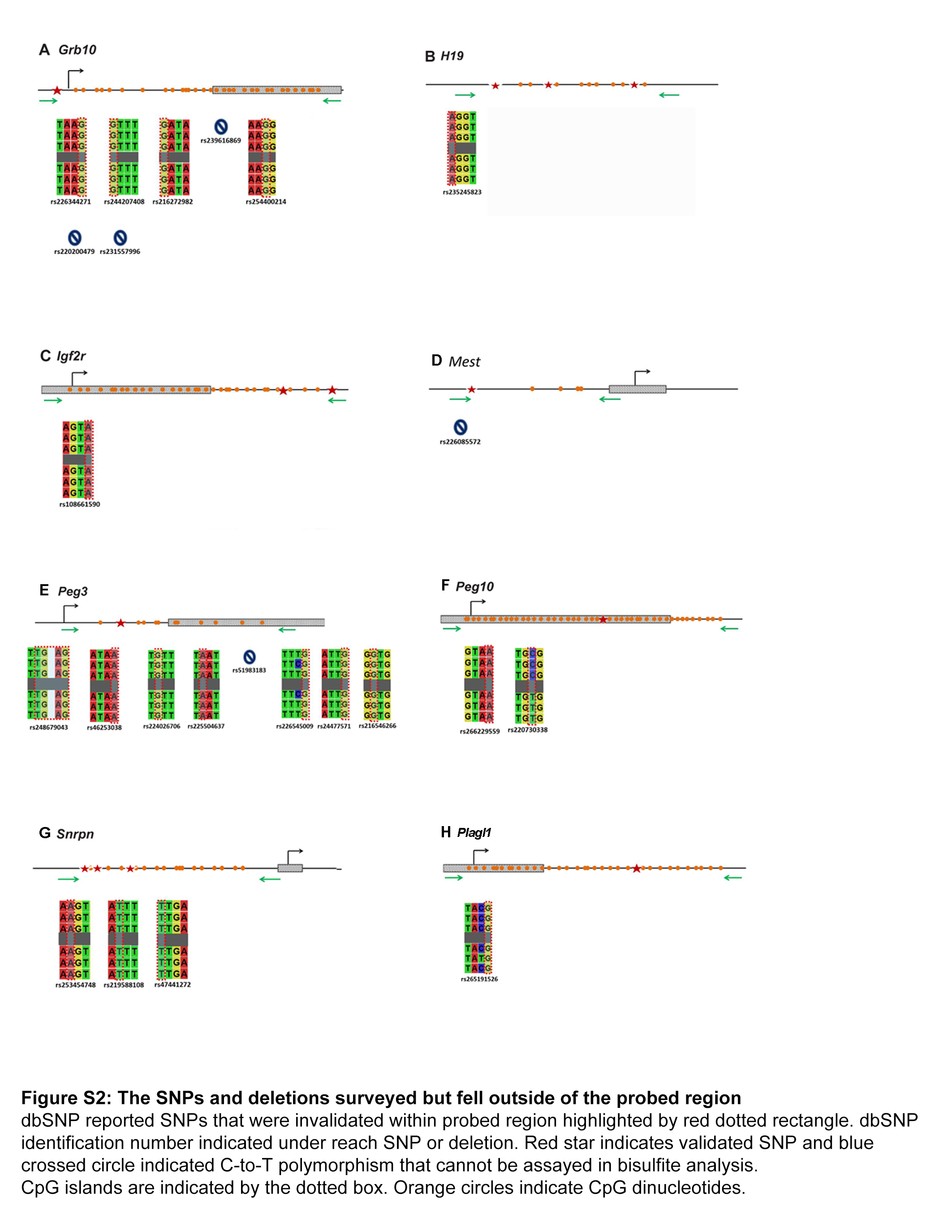

Supplement: Supplementary file 2 [file 91FigureS2.tif]
